# Cell membrane-bound toll-like receptor-1/2/4/6 monomers and -2 heterodimer inhibit enterovirus 71 replication by activating the antiviral innate response

**DOI:** 10.3389/fimmu.2023.1187035

**Published:** 2023-05-03

**Authors:** Ping-Ping Sun, Dan Li, Meng Su, Qing Ren, Wen-Ping Guo, Jiang-Li Wang, Luan-Ying Du, Guang-Cheng Xie

**Affiliations:** ^1^Department of Pathogenic Biology, College of Basic Medicine, Chengde Medical University, Chengde, Hebei, China; ^2^Department of Microbiology Laboratory, Chengde Center for Disease Control and Prevention, Chengde, Hebei, China; ^3^Institute of Basic Medicine, College of Basic Medicine, Chengde Medical University, Chengde, Hebei, China

**Keywords:** enterovirus 71, toll-like receptors, capsid proteins, antiviral innate immunity, immune activation

## Abstract

Host immune activation is critical for enterovirus 71 (EV71) clearance and immunopathogenesis. However, the mechanism of innate immune activation, especially of cell membrane-bound toll-like receptors (TLRs), against EV71 remains unknown. We previously demonstrated that TLR2 and its heterodimer inhibit EV71 replication. In this study, we systematically investigated the effects of TLR1/2/4/6 monomers and TLR2 heterodimer (TLR2/TLR1, TLR2/TLR6, and TLR2/TLR4) on EV71 replication and innate immune activation. We found that the overexpression of human- or mouse-derived TLR1/2/4/6 monomers and TLR2 heterodimer significantly inhibited EV71 replication and induced the production of interleukin (IL)-8 via activation of the phosphoinositide 3-kinase/protein kinase B (*PI3K/AKT*) and mitogen-activated protein kinase (*MAPK*) pathways. Furthermore,human–mouse chimeric TLR2 heterodimer inhibited EV71 replication and activated innate immunity. Dominant-negative TIR-less (DN)-TLR1/2/4/6 did not exert any inhibitory effects, whereas DN-TLR2 heterodimer inhibited EV71 replication. Prokaryotic expression of purified recombinant EV71 capsid proteins (VP1, VP2, VP3, and VP4) or overexpression of EV71 capsid proteins induced the production of IL-6 and IL-8 via activation of the *PI3K/AKT* and *MAPK* pathways. Notably, two types of EV71 capsid proteins served as pathogen-associated molecular patterns for TLR monomers (TLR2 and TLR4) and TLR2 heterodimer (TLR2/TLR1, TLR2/TLR6, and TLR2/TLR4) and activated innate immunity. Collectively, our results revealed that membrane TLRs inhibited EV71 replication via activation of the antiviral innate response, providing insights into the EV71 innate immune activation mechanism.

## 1 Introduction

Enterovirus 71 (EV71) is the major causative agent of hand, foot, and mouth disease (HFMD) that poses a heavy burden on affected infants and children under five years of age. EV71 was first identified in patients with fatal encephalitis in California, USA, in 1969 (1). In 1973, EV71 was recognized as the causative pathogen of HFMD, although it was also observed in patients with aseptic meningitis in Japan (2). Several HFMD outbreaks have occurred worldwide, particularly in Bulgaria in 1975 (3), Malaysia in 1997 (4), and Taiwan in 1998 (5). China also experienced large outbreaks of HFMD in Linyi in 2007 (6), Fuyang in 2008 (7), and Guangdong in 2009 (8). Circulating EV71 has also been reported in Asia-Pacific countries, including Japan, Singapore, Indonesia, Thailand, and the Philippines (9–11). Therefore, HFMD caused by EV71 infection is a serious threat to global public health. EV71 is a single-stranded positive-sense RNA virus belonging to the genus, *Enterovirus*, of the *Picornaviridae* family. The single open reading frame of the EV71 genome encodes only one polyprotein, which is further hydrolyzed and degraded by 2A and 3C proteases to form four structural proteins (VP1, VP2, VP3, and VP4) and seven non-structural proteins (2A, 2B, 2C, 3A, 3B, 3C, and 3D) (12, 13). Four capsid proteins form the icosahedral capsid of EV71. Structural proteins VP1, VP2, and VP3 are located on the surface, whereas VP4 is internalized in the EV71 capsid (11, 12). In the EV71 life cycle, binding of scavenger receptor class B member 2 (SCARB2) or selectin P ligand (SELPLG, also known as PSGL-1) with capsid proteins is a critical step in the EV71 attachment process (14, 15). All four capsid proteins of EV71 play different roles in the infection cycle. VP1 is mainly involved in receptor binding, viral entry, and virion assembly, and mutations at its amino acid residue 145 determine the virulence of EV71 (10, 16). During viral entry, EV71 VP4 protein plays an important role in the formation of pores in the cell membrane to release viral RNA into the host cell cytoplasm via myristoylation of VP4 to interact with the cell membrane (17).

Innate antiviral immunity is the first line of host defense against viral infections. Pattern recognition receptors (PRRs), including toll-like receptors (TLRs), retinoic acid-inducible gene I-like receptors, and nucleotide oligomerization domain-like receptors, recognize conserved viral RNA/DNA or proteins, known as pathogen-associated molecular patterns (PAMPs), to initiate the signaling cascade for the production of effector molecules (18, 19). TLRs are a class of type I transmembrane PRRs. To date, 10 and 12 TLRs have been identified in humans and mice, respectively. Human TLRs are further divided into two groups based on their membrane location: cell membrane-bound TLRs (TLR1, 2, 4, 5, 6, and 10), and endosomal or endolysosomal membrane-bound TLRs (TLR3, 7, 8, and 9) (20–22). Using EV71 viral RNA as a ligand, interferon induced with helicase C domain 1 activates the interferon (IFN) regulatory factor 3 (IRF3) and induces IFN-β expression (23). Transcriptional levels of TLR7 and TLR8 are significantly upregulated in EV71-infected HT29 cells (24) and human primary monocyte-derived macrophages (MDMs) (25).

Clinical samples, such as blood and cerebrospinal fluid samples, of EV71-infected patients exhibit significantly upregulated levels of IFN-γ-induced protein 10 (IP-10), monocyte chemoattractant protein-1 (MCP-1), monokine induced by IFN-γ, and interleukin (IL)-8 (26), and altered levels of proinflammatory cytokines (IL-1β, IL-2, IL-23, IL-33, and tumor necrosis factor [TNF]-α), chemokines (IP-10 and MCP-1), and other cytokines (IL-6, IL-8, IL-10, and IL-18) (27–29), indicating the activation of cellular immune responses and involvement of specific cytokines in the pathogenesis of EV71 infection. Therefore, it is necessary to determine the immune activation mechanism, especially of innate immunity, to understand the antiviral activity of host cells against EV71. Interestingly, EV71 exhibits an immune evasion strategy to avoid innate immunity using its 2A and 3C proteases by cleaving PRRs or adaptors, including TIR-domain-containing adapter-inducing interferon-β, mitochondrial antiviral-signaling protein, and IRF7, to block PRR recognition and inhibit signaling cascade transduction (10, 30). However, the specific innate immune activation mechanism of host cells against EV71 remains unknown. Antiviral innate immunity against EV71 mediated by PRRs sensing viral nucleotides in the cytoplasm is often disrupted. Whether host cells use cell membrane-bound TLRs, such as TLR2 or TLR4, to recognize the viral proteins of EV71 to activate innate immunity remains unclear.

TLR2 and TLR4 mainly recognize bacterial PAMPs, such as lipopolysaccharides and flagellins (31, 32). Various viral proteins, such as dengue virus NS1 protein (33), human immunodeficiency virus (HIV)-1 structural proteins (p17, p24, and pg41) (34), envelope gp120 glycoprotein (35), respiratory syncytial virus (RSV) G protein (36), influenza virus extracellular nucleoprotein (37), and other viral proteins (22, 38), are also recognized by TLR2 and TLR4. TLR2 expression levels are significantly upregulated in human rhinovirus 6 (HRV6)-infected or UV-inactivated HRV6-induced human airway epithelial cells (39). TLR2 expression levels are also significantly increased in EV71-infected or UV-inactivated EV71-induced MDMs (25). We previously reported that the transcriptional levels of TLR2 are upregulated in EV71-infected cells via transcriptomic sequencing (40). Moreover, we previously demonstrated that EV71 replication is significantly inhibited by the transfection of TLR2 or TLR2 heterodimer (TLR2/TLR1 and TLR2/TLR6) plasmids or activation of TLR2 heterodimer by Pam2CSK4 and Pam3CSK4 in HEK293 cells (40). However, the specific effects of TLR2, TLR4, and TLR2 heterodimer on EV71 remain unknown. To determine whether cell membrane-bound TLR1/2/4/6 monomers and TLR2 heterodimer exhibit any antiviral activity against EV71, we systematically investigated the replication of EV71, production of IL-8, and activation of the phosphoinositide 3-kinase/protein kinase B (*PI3K/AKT*) and mitogen-activated protein kinase (*MAPK*) pathways (extracellular signal-regulated kinase [*ERK*], c-Jun N-terminal kinase [*JNK*], and *p38* pathways) in HEK293 cells transfected with different TLR1/2/4/6 monomer and TLR2 heterodimer plasmids. We further evaluated the effects of EV71 capsid proteins on cytokine production and pathway activation using TLR monomers (TLR2 and TLR4) and TLR2 heterodimer (TLR2/TLR1, TLR2/TLR6, and TLR2/TLR4). Our findings revealed that EV71 recognition led to the activation of antiviral innate immunity to inhibit its replication via TLR1/2/4/6 monomers and TLR2 heterodimer. Moreover, we determined the roles of EV71 capsid proteins in the activation of TLR monomer (TLR2 and TLR4) and TLR2 heterodimer (TLR2/TLR1, TLR2/TLR6, and TLR2/TLR4) signaling.

## 2 Materials and methods

### 2.1 Cell lines and virus

Human embryonic kidney (HEK293) and rhabdomyosarcoma (RD) cells were cultured in Dulbecco’s modified Eagle’s medium (Gibco, CA, USA). The human tonsillar epithelial (UT-SCC-60B) cell line was kindly provided by Dr. Reidar Grénman and maintained in the Roswell Park Memorial Institute-1640 medium (Gibco). All media were supplemented with 10% bovine calf serum and 1% 100× penicillin–streptomycin (Biosharp, China). Cells were incubated at 37°C in a 5% CO_2_ humidified incubator.

EV71 (Fuyang strain; GenBank: EU703812.1) was used in this study and propagated in RD cells. Propagation and purification methods for EV71 were described in our previous study (40).

### 2.2 Plasmids

Human TLR plasmids, including pcDNA3-TLR1-YFP (Addgene plasmid #13014), pcDNA3-TLR2-CFP (Addgene plasmid #13015), pcDNA3-TLR4-YFP (Addgene plasmid #13018), and pcDNA3-TLR6-YFP (Addgene plasmid #13020), were gifts from Doug Golenbock. Mouse TLR plasmids, including mTLR1 (Addgene plasmid #13080), mTLR2 (Addgene plasmid #13083), and mTLR4 (Addgene plasmid #13085), were gifts from Ruslan Medzhitov. Point mutation plasmids of TLR4, including pMyc-CMV1-huTLR4mut-C1196T (Addgene plasmid #53526), pMyc-CMV1-huTLR4mut-C2141A (Addgene plasmid #53527), and pMyc-CMV1-huTLR4mut-A896G (Addgene plasmid #53525), were gifts from Linda Yu. All these plasmids were purchased from Addgene (https://www.addgene.org). mTLR6 plasmid pUNO1-mTLR06-HA3x (puno1ha-mtlr6), TLR2 heterodimer plasmids, including pDUO-hTLR6/TLR2 (pduo-htlr6tlr2), pDUO-hTLR1/TLR2 (pduo-htlr1tlr2), and pDUO-hCD14/TLR2 (pduo-hcd14tlr2) plasmids, TLR1/2/4/6 Dominant-negative TIR-less (DN [ΔTIR]) plasmids, pUNO1-hTLR1-DN-HA (puno1ha-htlr1-dn), pUNO1-hTLR2-DN-HA (puno1ha-htlr2-dn), pUNO1-hTLR4-DN-HA (puno1ha-htlr4a-dn), and pUNO1-hTLR6-DN-HA (puno1ha-htlr6-dn), were purchased from InvivoGen (San Diego, CA, USA).

Primers for EV71 structural proteins (VP1, VP2, VP3, and VP4) were designed based on the genome of the EV71 Fuyang strain (GenBank: EU703812.1). HindIII and NotI restriction enzyme sequences were added to forward and reverse primers, respectively. Viral RNA was extracted using the Viral Nucleic Acid Extraction Kit II (Geneaid, Taiwan), and cDNA was synthesized using the First-strand cDNA Synthesis Kit (Beyotime Biotechnology, Shanghai, China). Full-length genes of VP1, VP2, VP3, and VP4 were amplified using Easy-Load PCR Master Mix (Beyotime Biotechnology). Purified genes of VP1, VP2, VP3, VP4, and pcDNA3.1(+)/myc-His A vectors were digested using HindIII and NotI (New England Biolabs, Beijing, China) and ligated using T4 DNA ligase (New England Biolabs, Beijing, China). Recombinant plasmids pcDNA3.1-EV71-VP1, pcDNA3.1-EV71-VP2, pcDNA3.1-EV71-VP3, and pcDNA3.1-EV71-VP4 were confirmed via polymerase chain reaction (PCR) amplification.

### 2.3 Prokaryotic expression and purification

Full-length VP1, VP2, VP3, and VP4 segments of the EV71 Fuyang strain were codon-optimized and synthesized by Genewiz (Suzhou, China) and cloned into a pGEX4T-1 prokaryotic expression vector. Recombinant pGEX4T-EV71-VP1, pGEX4T-EV71-VP2, pGEX4T-EV71-VP3, and pGEX4T-EV71-VP4 plasmids were transformed into *Escherichia coli* strain BL21(DE3) (TianGen, Beijing, China). Recombinant EV71 VP1, VP2, VP3, and VP4 proteins were expressed with isopropyl-β-D-thiogalactopyranoside at a final concentration of 0.5 mM when the optical density (OD)_600_ of bacterial cultures reached 0.6–0.8. Bacterial sediment was collected and sonicated for 30 min after culture for 16 h at 22°C. The supernatant was centrifuged and filtered by 0.45 and 0.22 μm filter membranes to remove the bacterial debris and mixed with glutathione S-transferase (GST)-tag purification resin (Beyotime Biotechnology) for 2 h at room temperature. GST fusion proteins were collected with an elution buffer (10 mM reduced glutathione, 50 mM Tris-HCl, pH8.0) after washing five times with 30 mL phosphate-buffered saline (PBS; pH7.0). Purified GST fusion proteins were detected using sodium dodecyl sulfate-polyacrylamide gel electrophoresis (SDS-PAGE) and their concentrations were determined using a BCA kit (Beyotime Biotechnology), according to the manufacturer’s instructions.

### 2.4 Transfection and infection

Endotoxin-free TLR-related and EV71 capsid recombinant eukaryotic plasmids were prepared using the E.Z.N.A. Endo-free Plasmid Kit (OMEGA, USA), according to the manufacturer’s instructions. HEK293 and RD cells were seeded in a 6-well plate and the TLR-related or EV71 capsid recombinant eukaryotic plasmids were transiently single or co-transfected into host cells at the indicated dose (1 or 2 μg) using the SuperFect Transfection Reagent (Qiagen, Germany), according to the manufacturer’s instructions.

After transfecting the plasmids into cells for 24 h, TLR-overexpressing HEK293 and RD cells were infected with EV71 at a multiplicity of infection (MOI) of 0.5 or 1. Then, cytopathic effects (CPEs) on non-transfected and transfected HEK293 and RD cells were observed 24 h after EV71 infection using a microscope (Olympus, Japan).

### 2.5 Real-time polymerase chain reaction

EV71 viral RNA was extracted from the supernatant using a Viral Nucleic Acid Extraction Kit II (Geneaid) according to the manufacturer’s instructions. Reverse transcription was performed using the First-strand cDNA Synthesis Kit (Beyotime Biotechnology) to synthesize cDNA. Real-time polymerase chain reaction (PCR) was performed with the MyiQ2 Real-Time PCR system (Bio-Rad, California, USA) using 2×TransStart Top Green qPCR SuperMix (Trans, Beijing, China) according to manufacturer’s instructions. Primers and reaction conditions for real-time PCR were as described in our previous study (40).

### 2.6 Western blot

Cell lysates were prepared using the radioimmunoprecipitation assay lysis buffer (Beyotime Biotechnology) supplemented with 50× protease and phosphatase inhibitor cocktail (Beyotime Biotechnology). Then, cell lysates were centrifuged to remove the cellular debris and boiled at 100°C for 5 min. All prepared samples were subjected to SDS-PAGE with 12% separating gel, transferred to 0.2-μm PVDF membrane (Merck Millipore, Carrigtwohill, Ireland), and blocked with 5% skim milk in PBST for 1.5 h at room temperature. Primary antibodies were diluted 1:1000 in PBST containing 5% bovine serum albumin. The blocked PVDF membrane was incubated with the diluted primary antibodies at 4°C overnight, followed by incubation with the diluted secondary antibodies for 2 h at room temperature after washing thrice with PBST for 10 min. Finally, the membranes were incubated with an ECL detection kit (Biosharps), and the protein bands were visualized using a Tanon 6100 system (Tanon, Shanghai, China).

The following primary antibodies were used in this study: PI3K p85 (Cell Signaling, Danvers, MA, USA), p-AKT (Ser473; Cell Signaling), p-ERK1/2 (Thr202/Tyr204; Cell Signaling), p-JNK (Thr183/Tyr185; Cell Signaling), p-p38 (Thr180/Tyr182; Cell Signaling), TLR2 (Abcam, Cambridge, MA, USA), TLR1 (Abcam), TLR6 (Cell Signaling), His (Trans), p-PI3K p85α/β/p55γ (Y467/Y464/Y199; Beyotime Biotechnology), and β-actin (Beyotime Biotechnology).

### 2.7 Enzyme-linked immunosorbent assay

The supernatant was collected from EV71-infected plasmid non-transfected and transfected HEK293 and RD cells. UT-SCC-60B cells were stimulated with recombinant EV71 capsid proteins at a final concentration of 80 μg/mL and single transfected with EV71 capsid recombinant eukaryotic plasmids or co-transfected with TLR-related and EV71 capsid recombinant eukaryotic plasmids. HEK293 cells were transfected with TLR-related plasmids for 24 h and stimulated with recombinant EV71 capsid proteins at a final concentration of 80 μg/mL. Supernatants from the above experiments were collected. Levels of IL-6 and IL-8 in the supernatants were measured using the Human IL-6 and IL-8 ELISA Kits (Beyotime Biotechnology), according to the manufacturer’s instructions.

### 2.8 Statistical analysis

All experiments were conducted in triplicate. Data are represented as the mean ± standard deviation. Statistical analysis was conducted using IBM SPSS Statistics software (version 19.0; IBM Corp., USA). Student’s *t*-test was used to determine the statistical differences between the two groups. One-way analysis of variance was used to determine the statistical differences among three or more groups. Differences were considered statistically significant at p < 0.05.

## 3 Results

### 3.1 Human- or mouse-derived TLR1/2/6 monomers and TLR2 heterodimer inhibit replication of and activate innate immunity against EV71

**Figure 1:**
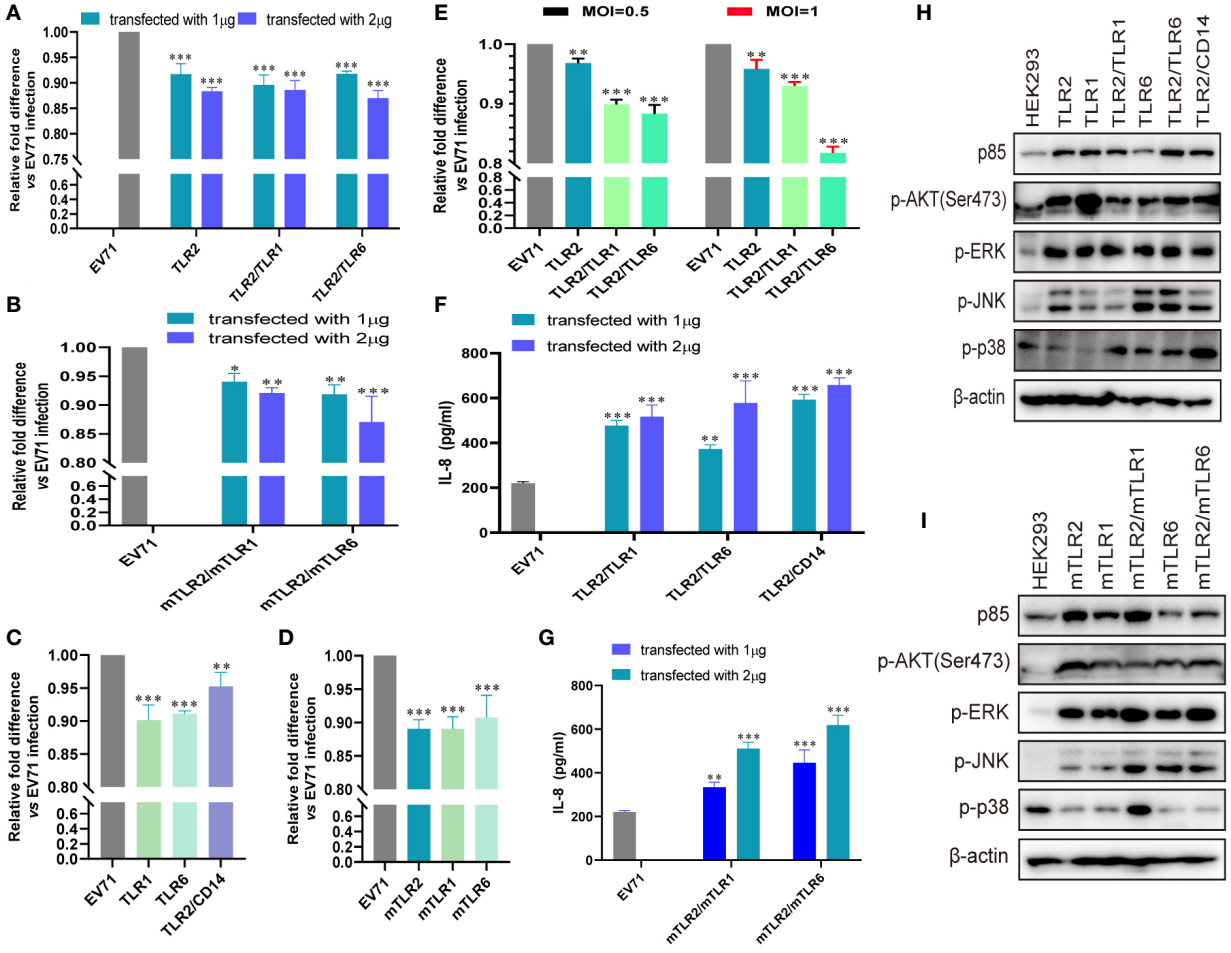
EV71 infection activates the antiviral innate immunity via toll-like receptor (TLR) monomers and TLR2 heterodimers to limit its replication. **(A)** Human-derived TLR2 and TLR2 heterodimer (TLR2/TLR1 and TLR2/TLR6) or **(B)** mouse-derived TLR2 heterodimer (mTLR2/mTLR1 and mTLR2/mTLR6) plasmids were transfected into HEK293 cells at a dose of 1 or 2 μg for 24 h, followed by infection with EV71 at a multiplicity of infection (MOI) of 1 for 24 (h) n = 3. **(C)** Human-derived TLR1, TLR6, and TLR2/CD14 or **(D)** mouse-derived mTLR2, mTLR1, and mTLR6 plasmids were transfected into HEK293 cells at a dose of 1 μg for 24 h, followed by infection with EV71 at an MOI of 1 for 24 (h) **(E)** Human-derived TLR2 and TLR2 heterodimer (TLR2/TLR1 and TLR2/TLR6) plasmids were transfected into rhabdomyosarcoma (RD) cells for 24 h and infected with EV71 at an MOI of 0.5 or 1 for 24 (h) Genome copies of EV71 were determined using quantitative polymerase chain reaction (qPCR), and relative fold differences compared with HEK293 cells infected with EV71 for 24 h were calculated. n = 3. **(F)** Human-derived TLR2 heterodimer (TLR2/TLR1, TLR2/TLR6, and TLR2/CD14) or **(G)** mouse-derived TLR2 heterodimer (mTLR2/mTLR1 and mTLR2/mTLR6) plasmids were transfected into HEK293 cells at a dose of 1 or 2 μg for 24 h, followed by infection with EV71 at an MOI of 1 for 24 (h) Supernatants were collected and interleukin (IL)-8 concentrations were determined using an enzyme-linked immunosorbent assay (ELISA) kit. n = 3. **(H)** Human-derived TLR monomer (TLR2, TLR1, and TLR6) and TLR2 heterodimer (TLR2/TLR1, TLR2/TLR6, and TLR2/CD14) plasmids or **(I)** mouse-derived TLR monomer (mTLR2, mTLR1, and mTLR6) and TLR2 heterodimer (mTLR2/mTLR1 and mTLR2/mTLR6) plasmids were transfected into HEK293 cells at a dose of 1 μg for 24 h, followed by infection with EV71 at an MOI of 1 for 24 (h) Total proteins were collected, and activation of the phosphoinositide 3-kinase/protein kinase B (*PI3K/AKT*) and mitogen-activated protein kinase (*MAPK*) pathways was assessed via western blotting. n = 3. *p < 0.05, **p < 0.01, and ***p < 0.001.

Previously, we demonstrated that TLR2 and TLR2 heterodimer reduce CPEs and EV71 replication in TLR2 and TLR2 heterodimer-overexpressing cells (40). However, we could not determine the specific mechanism of activation of antiviral innate immunity against EV71 via cell membrane-bound TLRs. To determine the roles of cell membrane-bound TLRs, such as TLR1/2/4/6 monomers and TLR2 heterodimer, in EV71 replication and innate immunity activation, we first transfected human-derived TLR1/2/6 monomers and TLR2 heterodimer (TLR2/TLR1 and TLR2/TLR6) into HEK293 cells and then infected them with EV71 in this study. EV71 replication was significantly and dose-dependently decreased in TLR2 and TLR2 heterodimer (TLR2/TLR1 and TLR2/TLR6)-overexpressing HEK293 cells (**Figure 1A**). To determine whether mouse-derived TLR2 heterodimer (mTLR2/mTLR1 and mTLR2/mTLR6) inhibit EV71 replication, mouse-derived TLR2 heterodimer (mTLR2/mTLR1 and mTLR2/mTLR6) were overexpressed in HEK293 cells. EV71 replication was inhibited in a dose-dependent manner (**Figure 1B**). To determine whether TLR1 and TLR6 monomers could inhibit EV71 replication, we transfected human- or mouse-derived TLR1 and TLR6 into HEK293 cells. EV71 replication was significantly decreased in human-derived TLR1- and TLR6-overexpressing HEK293 cells; the TLR2/CD14 heterodimer also decreased EV71 replication (**Figure 1C**). Similar results were observed in the mTLR1-, mTLR2-, and mTLR6-overexpressing cells (**Figure 1D**). Levels of the EV71 genome were also significantly decreased in RD cells overexpressing human-derived TLR2 and TLR2 heterodimer (TLR2/TLR1 and TLR2/TLR6) at different MOI of EV71 infection, especially the TLR2/TLR6 heterodimer (**Figure 1E**).

EV71 replication is significantly inhibited by cell membrane-bound TLR1/2/6 and TLR2 heterodimer (TLR2/TLR1, TLR2/TLR6, and TLR2/CD14). Given that innate antiviral immunity is the first line of defense against viral infections, we hypothesized that EV71 activates innate immunity via TLR2 heterodimer recognition. First, we confirmed that IL-8 production was significantly upregulated in human TLR2 heterodimer (TLR2/TLR1, TLR2/TLR6, and TLR2/CD14; **Figure 1F**) or mouse-derived TLR2 heterodimer (mTLR2/mTLR1 and mTLR2/mTLR6; **Figure 1G**) overexpressing HEK293 cells upon EV71 infection for 24h and appeared in a dose-dependent manner. Second, we investigated whether inflammatory pathways were activated in TLR-overexpressing cells following EV71 infection. Expression of PI3K regulatory subunit p85 was upregulated and phosphorylation levels of AKT, ERK, JNK, and p38 were also increased at different levels in cells overexpressing human-derived TLR monomers (TLR1, TLR2, and TLR6) and TLR2 heterodimer (TLR2/TLR1, TLR2/TLR6, and TLR2/CD14; **Figure 1H**) or in those overexpressing mouse-derived TLR monomers (mTLR2, mTLR1, and mTLR6) and TLR2 heterodimer (mTLR2/mTLR1, mTLR2/mTLR6; **Figure 1I**). These results suggest that TLR monomers and TLR2 heterodimer play important roles in inhibiting EV71 replication and activating innate immunity against EV71.

### 3.2 Conditional changes in TLR2 heterodimer inhibit the replication of and activate innate immunity against EV71

**Figure 2:**
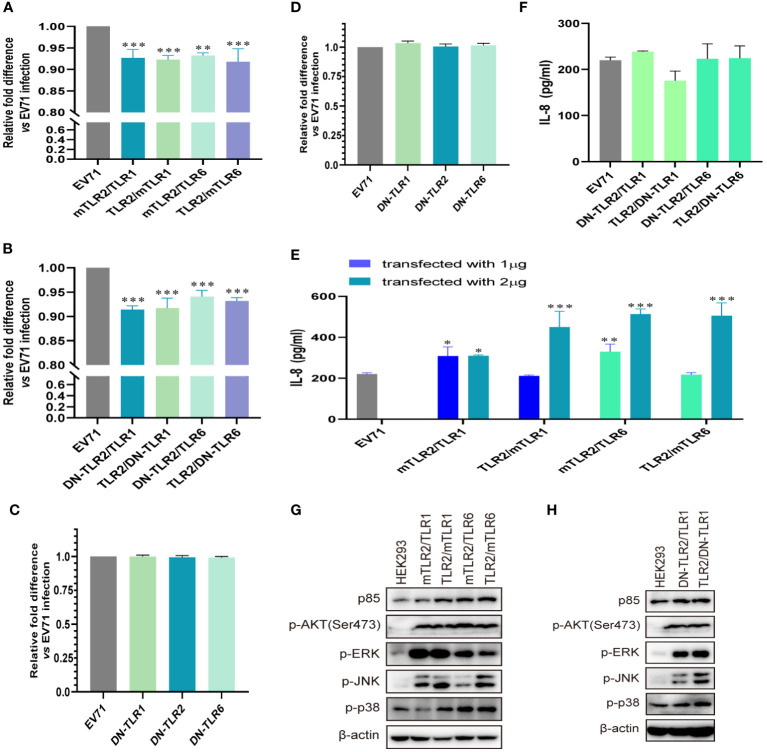
EV71 replication is inhibited by conditional changes in TLR2 heterodimers. **(A)** Human–mouse chimeric TLR2 heterodimer (mTLR2/TLR1, TLR2/mTLR1, mTLR2/TLR6, and TLR2/mTLR6) plasmids and **(B)** single dominant-negative TIR-less (DN) TLR2 heterodimer (DN-TLR2/TLR1, TLR2/DN-TLR1, DN-TLR2/TLR6, and TLR2/DN-TLR6) plasmids were transfected into HEK293 cells at a dose of 1 μg for 24 h, followed by infection with EV71 at an MOI of 1 for 24 (h) DN-TLR (DN-TLR1, DN-TLR2, and DN-TLR6) plasmids were transfected into **(C)** HEK293 or **(D)** UT-SCC-60B cells at a dose of 1 μg for 24 h, followed by infection with EV71 at an MOI of 1 for 24 (h) n = 3. Genome copies of EV71 were determined and relative fold differences were calculated. **(E)** Human–mouse chimeric TLR2 heterodimer (mTLR2/TLR1, TLR2/mTLR1, mTLR2/TLR6, and TLR2/mTLR6) plasmids were transfected into HEK293 cells at a dose of 1 or 2 μg for 24 h, followed by infection with EV71 at an MOI of 1 for 24 (h) n = 3. **(F)** Concentrations of IL-8 in the supernatant of group **(B)**. n = 3. **(G)** Human–mouse chimeric TLR2 heterodimer (mTLR2/TLR1, TLR2/mTLR1, mTLR2/TLR6, and TLR2/mTLR6) plasmids or **(H)** single dominant-negative TIR-less TLR2 heterodimer (DN-TLR2/TLR1 and TLR2/DN-TLR1) plasmids were transfected into HEK293 cells at a dose of 1 μg for 24 h, followed by infection with EV71 at an MOI of 1 for 24 (h) Total proteins were collected, and activation of the *PI3K/AKT* and *MAPK* pathways was assessed via western blotting. n = 3. *p < 0.05, **p < 0.01, and ***p < 0.001.

To further determine whether TLR1/2/6 monomers and TLR2 heterodimer play important roles in activating antiviral innate immunity against EV71, we investigated whether conditional changes in TLR2 heterodimer affect the inhibition of EV71 replication and activation of innate immunity. Overexpression of human–mouse chimeric TLR2 heterodimer (mTLR2/TLR1, TLR2/mTLR1, mTLR2/TLR6, and TLR2/mTLR6) significantly inhibited EV71 replication (**Figure 2A**), and EV71 replication was also inhibited in DN-TLR2 heterodimer (DN-TLR2/TLR1, TLR2/DN-TLR1, DN-TLR2/TLR6, TLR2/DN-TLR6)-overexpressing HEK293 cells (**Figure 2B**). However, EV71 replication was not inhibited in DN-TLRs (DN-TLR1, DN-TLR2, and DN-TLR6)-overexpressing HEK293 cells (**Figure 2C**) and UT-SCC-60B cells (**Figure 2D**). Levels of IL-8 were also significantly upregulated in cells overexpressing human–mouse chimeric TLR2 heterodimer (mTLR2/TLR1, TLR2/mTLR1, mTLR2/TLR6, and TLR2/mTLR6), especially mTLR2/TLR6 heterodimer, in a dose-dependent manner (**Figure 2E**); however, levels of IL-8 were not upregulated in DN-TLR2 heterodimer (DN-TLR2/TLR1, TLR2/DN-TLR1, DN-TLR2/TLR6, and TLR2/DN-TLR6; **Figure 2F**) and DN-TLR monomers (DN-TLR1, DN-TLR2, and DN-TLR6; **Figure S1A**). Expression levels of p85 and phosphorylation levels of AKT, ERK, JNK, and p38 were upregulated in human–mouse chimeric TLR2 heterodimer (mTLR2/TLR1, TLR2/mTLR1, mTLR2/TLR6, and TLR2/mTLR6; **Figure 2G**) and DN-TLR2 heterodimer (DN-TLR2/TLR1, TLR2/DN-TLR1)-overexpressing HEK293 cells (**Figure 2H**); however, the opposite results were obtained with DN-TLR monomers (DN-TLR1, DN-TLR2, and DN-TLR6; **Figure S1B**). These findings confirmed that TLR monomers (TLR1, TLR2, and TLR6) and TLR2 heterodimer (TLR2/TLR1 and TLR2/TLR6) play important roles in inhibiting EV71 replication and activating innate antiviral immunity against EV71.

### 3.3 Human- and mouse-derived TLR4 and TLR2/TLR4 heterodimer inhibit the replication of and activate innate immunity against EV71

**Figure 3:**
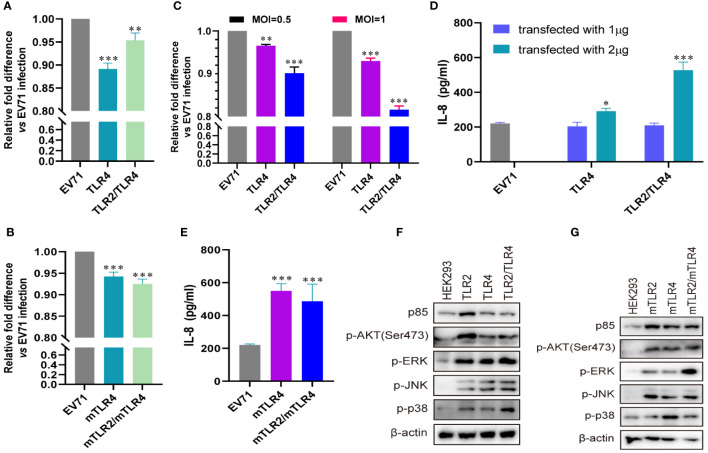
EV71 infection activates antiviral innate immunity via TLR4 and TLR2/TLR4 to limit its replication. **(A)** Human-derived TLR4 and TLR2/TLR4 plasmids or **(B)** mouse-derived mTLR4 and mTLR2/mTLR4 plasmids were transfected into HEK293 cells at a dose of 1 μg for 24 h, followed by infection with EV71 at an MOI of 1 for 24 (h) n = 3. **(C)** Human-derived TLR4 and TLR2/TLR4 plasmids were transfected into RD cells at a dose of 1 μg for 24 h, followed by infection with EV71 at an MOI of 0.5 or 1 for 24 (h) n = 3. Genome copies of EV71 were determined and relative fold differences were calculated. **(D)** Human-derived TLR4 and TLR2/TLR4 plasmids were transfected into HEK293 cells at a dose of 1 or 2 μg for 24 h, followed by infection with EV71 at an MOI of 1 for 24 (h) n = 3. **(E)** Concentrations of IL-8 in the supernatant of group **(B)** were determined. **(F)** Human-derived TLR2, TLR4, and TLR2/TLR4 plasmids or **(G)** mouse-derived mTLR2, mTLR4, and mTLR2/mTLR4 plasmids were transfected into HEK293 cells at a dose of 1 μg for 24 h, followed by infection with EV71 at an MOI of 1 for 24 (h) n = 3. Total proteins were collected, and activation of the *PI3K/AKT* and *MAPK* pathways was assessed via western blotting. *p < 0.05, **p < 0.01, and ***p < 0.001.

Influenza virus extracellular nucleoprotein and HIV-1 gp120 interact with TLR4 to mediate cytokine induction (35, 37). TLR2 interacts with TLR4 to form a novel TLR2/TLR4 heterodimer via hemoglobin (41). As TLR4 recognizes viral proteins to activate innate immunity, we evaluated the roles of TLR4 and TLR2/TLR4 heterodimer in EV71 infection. EV71 replication was significantly decreased in human-derived (**Figure 3A**) and mouse-derived (**Figure 3B**) TLR4 and TLR2/TLR4 heterodimer-overexpressing HEK293 cells. Furthermore, EV71 replication was significantly decreased in human-derived TLR4 and TLR2/TLR4 heterodimer-overexpressing RD cells in an infection-dose-dependent manner (**Figure 3C**). To assess IL-8 secretion in TLR4-and TLR2/TLR4 heterodimer-overexpressing cells upon EV71 infection, supernatants were collected and IL-8 levels were determined. Upregulation of IL-8 secretion was observed in human-derived TLR4 and TLR2/TLR4 heterodimer-overexpressing cells at a transfection dose of 2 μg (**Figure 3D**), and induction of IL-8 was also upregulated in mouse-derived TLR4 and TLR2/TLR4 heterodimer-overexpressing cells (**Figure 3E**). Expression levels of p85 and phosphorylation levels of AKT, ERK, JNK, and p38 were upregulated in human-derived (**Figure 3F**) and mouse-derived (**Figure 3G**) TLR2, TLR4, and TLR2/TLR4 heterodimer-overexpressing HEK293 cells. These data indicate that innate immunity is activated via the cell membrane-bound TLR4 monomer and that the TLR2/TLR4 heterodimer recognize EV71 to limit EV71 replication.

### 3.4 Conditional changes in TLR4 and TLR2/TLR4 heterodimer inhibit the replication of and activate innate immunity against EV71

**Figure 4:**
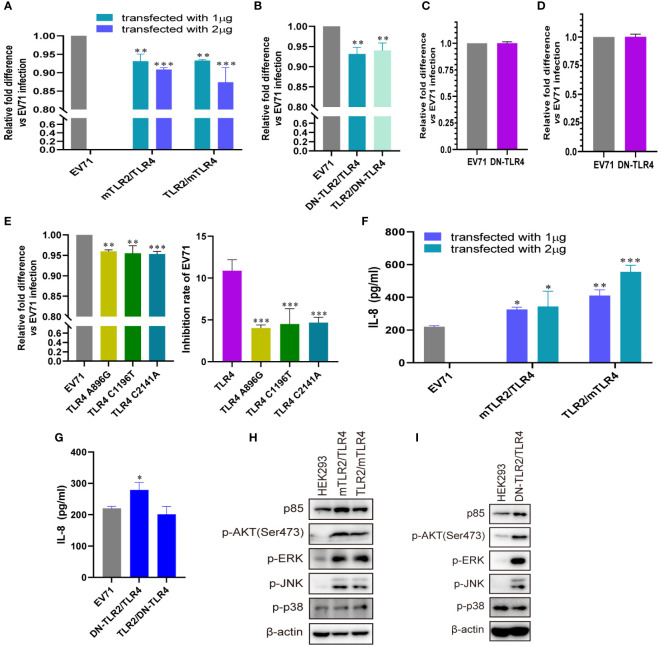
Conditional changes in TLR4 and TLR2/TLR4 heterodimers inhibit EV71 replication by activating innate immunity. **(A)** Human–mouse chimeric TLR2 heterodimer (mTLR2/TLR4 and TLR2/mTLR4) plasmids were transfected into HEK293 cells at a dose of 1 or 2 μg for 24 h, followed by infection with EV71 at an MOI of 1 for 24 (h) n = 3. **(B)** Single dominant-negative TIR-less TLR2/TLR4 heterodimer (DN-TLR2/TLR4 and TLR2/DN-TLR4) plasmids were transfected into HEK293 cells at a dose of 1 μg for 24 h, followed by infection with EV71 at an MOI of 1 for 24 **(h)** Single dominant-negative TIR-less TLR4 plasmid was transfected into **(C)** HEK293 or **(D)** UT-SCC-60B cells at a dose of 1 μg for 24 h, followed by infection with EV71 at an MOI of 1 for 24 (h) n = 3. **(E)** TLR4 single nucleotide mutation plasmids (A896G, C1196T, and C2141A) were transfected into HEK293 cells at a dose of 1 μg for 24 h, followed by infection with EV71 at an MOI of 1 for 24 (h) n = 3. Genome copies of EV71 were determined and relative fold differences were calculated. Concentrations of IL-8 in the supernatants of **(F)** human–mouse chimeric TLR2 heterodimer and **(G)** single dominant-negative TIR-less TLR2 heterodimer groups were determined. n = 3. Activation of the *PI3K/AKT* and *MAPK* pathways in **(H)** human–mouse chimeric TLR2 heterodimers and **(I)** DN-TLR2/TLR4 groups was assessed via western blotting. *p < 0.05, **p < 0.01, and ***p < 0.001.

To further confirm the roles of TLR4 and TLR2/TLR4 heterodimer in inhibiting EV71 replication and activating innate immunity, we investigated whether conditional changes in TLR4 and TLR2/TLR4 heterodimer affect EV71 replication and activation of innate immunity. When human–mouse chimeric TLR2/TLR4 heterodimer (mTLR2/TLR4 and TLR2/mTLR4) were overexpressed in HEK293 cells and infected with EV71, the replication of EV71 was significantly decreased in a dose-dependent manner (**Figure 4A**). We further determined that single dominant-negative TIR-less TLR2/TLR4 heterodimer (DN-TLR2/TLR4, TLR2/DN-TLR4) inhibited EV71 replication (**Figure 4B**); however, EV71 replication was not affected in DN-TLR4-overexpressing HEK293 (**Figure 4C**) and UT-SCC-60B (**Figure 4D**) cells. To identify the nucleotide acting as the major functional site for TLR4, three single nucleotide mutation TLR4 (A896G, C1196T, and C2141A) plasmids were transfected into HEK293 cells to determine their effects on EV71 replication. Mutations in TLR4 at nucleotides A896G, C1196T, and C2141A result in single amino acid mutations in TLR4 (Asp299Gly, Thr399Ile, and Pro714His, respectively) (42). We found that EV71 replication was also inhibited in HEK293 cells overexpressing TLR4 mutants (A896G, C1196T, and C2141A); however, the inhibition rates of these three TLR4 mutants were significantly lower than those of wild-type TLR4 (**Figure 4E**). Level of IL-8 secretion was significantly upregulated in HEK293 cells overexpressing human–mouse chimeric TLR2/TLR4 heterodimer (mTLR2/TLR4 and TLR2/mTLR4) in a dose-dependent manner (**Figure 4F**); however, only DN-TLR2/TLR4, but not TLR2/DN-TLR4 (**Figure 4G**) and DN-TLR4 (**Figure S1A**), upregulated IL-8 levels upon EV71 infection. *PI3K/AKT* and *MAPK* (ERK, JNK, and p38) pathways were also activated, with an increase in the phosphorylation levels of AKT, ERK, JNK, and p38 in human–mouse chimeric TLR2/TLR4 heterodimer (mTLR2/TLR4 and TLR2/mTLR4; **Figure 4H**) and DN-TLR2/TLR4 (**Figure 4I**). Expression levels of p85 and phosphorylation levels of AKT, ERK, and p38 were decreased in UT-SCC-60B overexpressing DN-TLR4 (**Figure S1B**). Taken together, these results further support the hypothesis that the TLR4 monomer and TLR2/TLR4 heterodimer inhibit EV71 replication by recognizing EV71 to activate innate immunity against it.

### 3.5 EV71 capsid proteins activate innate immunity via TLR2 and TLR2 heterodimer

**Figure 5:**
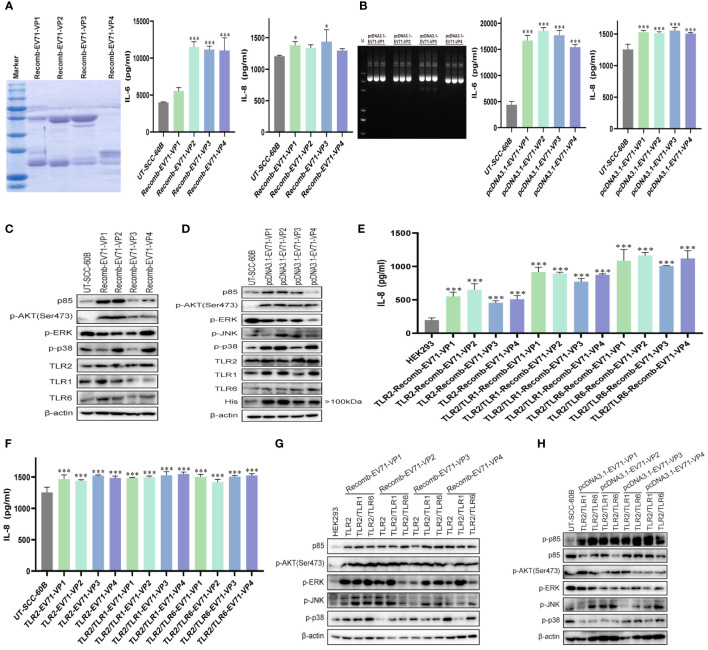
EV71 capsid proteins induce cytokine responses via TLR2 and TLR2 heterodimers. UT-SCC-60B cells were stimulated with purified recombinant prokaryotic-expressed EV71 capsid proteins at a final concentration of 80 μg/mL, or EV71 capsid recombinant eukaryotic plasmids were transfected into UT-SCC-60B cells at a dose of 1 μg for 24 (h) n = 3. Concentrations of IL-6 and IL-8 in **(A)** recombinant EV71 capsid protein stimulated and **(B)** EV71 capsid plasmid transfected groups were determined. Activation of the *PI3K/AKT* and *MAPK* pathways in **(C)** recombinant EV71 capsid protein stimulated and **(D)** EV71 capsid plasmid transfected groups were assessed via western blotting. Human-derived TLR2 and TLR2 heterodimer (TLR2/TLR1 and TLR2/TLR6) plasmids were transfected into HEK293 cells at a dose of 1 μg for 24 h and stimulated with recombinant EV71 capsid proteins at a final concentration of 80 μg/mL for 24 (h) n = 3. **(E)** Concentrations of IL-8 and **(G)** activation of the *PI3K/AKT* and *MAPK* pathways were determined. Human-derived TLR2 and TLR2 heterodimer (TLR2/TLR1 and TLR2/TLR6) and EV71 capsid plasmids were co-transfected into UT-SCC-60B cells at a dose of 1 μg for 24 (h) n = 3. **(F)** Concentrations of IL-8 and **(H)** activation of the *PI3K/AKT* and *MAPK* pathways were determined. *p < 0.05, **p < 0.01, and ***p < 0.001.

TLR2 expression is upregulated in response to live or UV-inactivated HRV6 and EV71 infection (25, 39, 40), suggesting that the enterovirus capsid may be recognized by TLR2. To determine whether EV71 capsid proteins activate cytokine response, we obtained purified prokaryotic expression recombinant EV71 capsid proteins VP1, VP2, VP3, and VP4 and used them to stimulate UT-SCC-60B cells at a final concentration of 80 μg/mL. IL-6 levels were significantly upregulated by recombinant EV71 VP2, VP3, and VP4, whereas IL-8 levels were significantly upregulated by recombinant EV71 VP1 and VP3 (**Figure 5A**). We next constructed EV71 capsid recombinant eukaryotic plasmids and transfected them into UT-SCC-60B cells. Levels of IL-6 and IL-8 were significantly upregulated in UT-SCC-60B cells overexpressing EV71 VP1, VP2, VP3, and VP4 (**Figure 5B**). Expression levels of p85, TLR1, TLR2, and TLR6 were upregulated by the recombinant EV71 capsid proteins VP1 and VP2, and the phosphorylation levels of ERK and p38 were significantly upregulated by recombinant EV71 capsid protein VP4 (**Figure 5C**). Expression levels of p85, TLR1, TLR2, and TLR6 and the phosphorylation levels of AKT, ERK, and p38 were upregulated in US-SCC-60B cells overexpressing EV71 capsid proteins at different levels (**Figure 5D**).

To determine whether EV71 capsid proteins activate innate immunity via TLR2 and TLR2 heterodimer (TLR2/TLR1 and TLR2/TLR6), we first transfected TLR2 and TLR2 heterodimer (TLR2/TLR1 and TLR2/TLR6) plasmids into HEK293 cells then stimulated with recombinant EV71 capsid proteins VP1, VP2, VP3, and VP4 at a final concentration of 80 μg/mL. IL-8 levels were significantly upregulated in TLR2 and TLR2 heterodimer (TLR2/TLR1 and TLR2/TLR6)-overexpressing HEK293 in response to stimulation with recombinant EV71 capsid proteins (VP1, VP2, VP3, and VP4), especially for the TLR2/TLR6 heterodimer (**Figure 5E**). Similar results were observed in TLR2 and TLR2 heterodimer (TLR2/TLR1 and TLR2/TLR6)-overexpressing UT-SCC-60B cells, in which EV71 capsid proteins (VP1, VP2, VP3, and VP4) were also overexpressed, and IL-8 levels were significantly upregulated (**Figure 5F**). *PI3K/AKT* and *MAPK* (ERK, JNK, and p38) pathways were activated at different levels by the recombinant EV71 capsid proteins via TLR2 and TLR2 heterodimer (TLR2/TLR1 and TLR2/TLR6), particularly EV71 VP1 (**Figure 5G**). These pathways were also activated in TLR2 heterodimer (TLR2/TLR1 and TLR2/TLR6) and EV71 capsid protein-overexpressing UT-SCC-60B cells (**Figure 5H**). Altogether, these results indicate that EV71 capsid proteins activate the *PI3K/AKT* and *MAPK* pathways via TLR2 and TLR2 heterodimer (TLR2/TLR1 and TLR2/TLR6), thereby increasing the production of pro-inflammatory cytokines and activating innate immunity.

### 3.6 EV71 capsid proteins activate innate immunity via TLR4 and TLR2/TLR4 heterodimer

**Figure 6:**
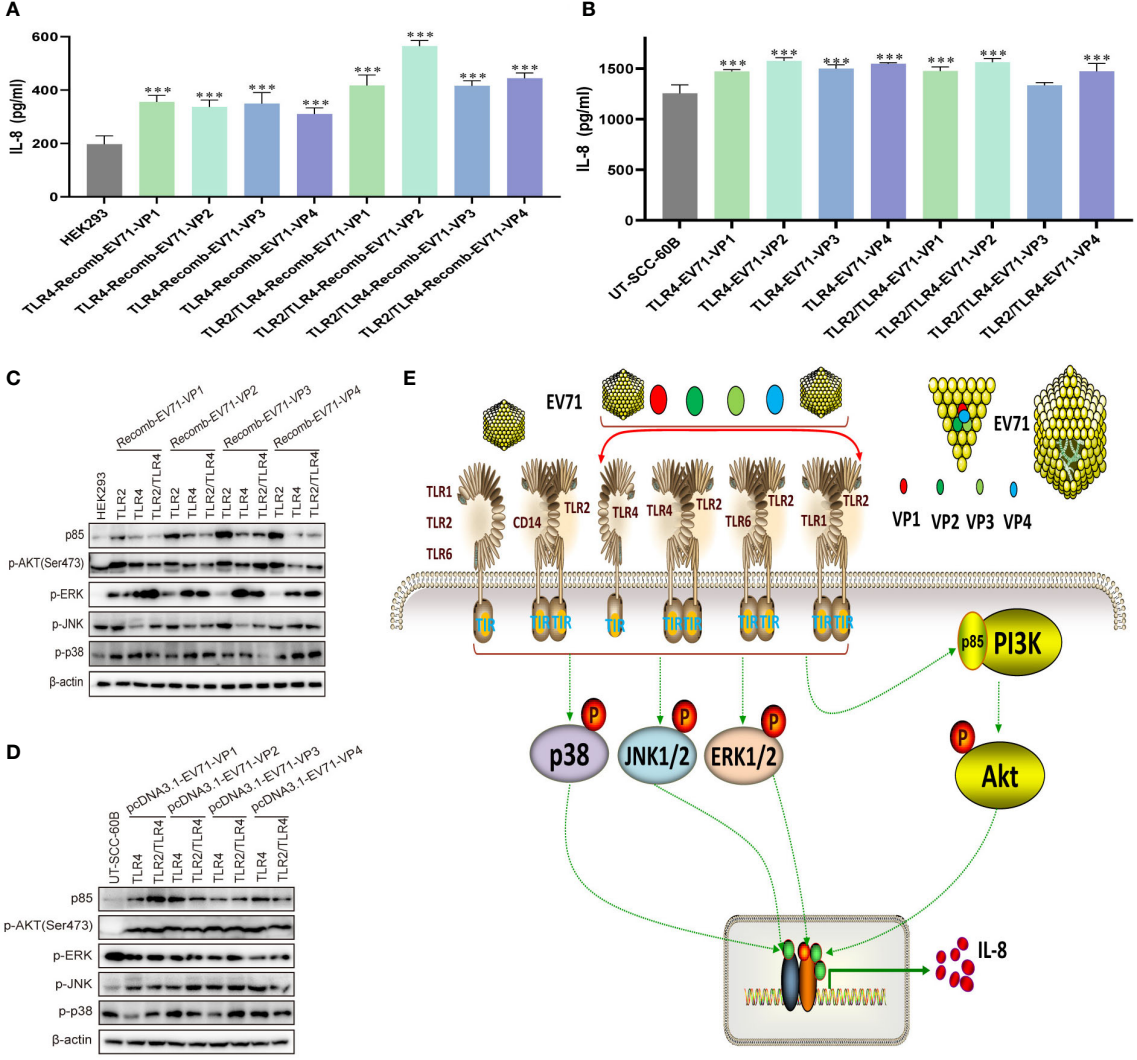
EV71 capsid proteins induce cytokine responses via TLR4 and TLR2/TLR4 heterodimer. Human-derived TLR4 and TLR2/TLR4 plasmids were transfected into HEK293 cells at a dose of 1 μg for 24 h, followed by stimulation with recombinant EV71 capsid proteins at a final concentration of 80 μg/mL for 24 (h) n = 3. **(A)** Concentrations of IL-8 and **(C)** activation of the *PI3K/AKT* and *MAPK* pathways were determined. Human-derived TLR4 and TLR2/TLR4 and EV71 capsid plasmids were co-transfected into UT-SCC-60B cells at a dose of 1 μg for 24 (h) n = 3. **(B)** Concentrations of IL-8 and **(D)** activation of the *PI3K/AKT* and *MAPK* pathways were determined. **(E)** Schematic model for the activation of innate immunity by EV71 and capsid proteins via cell membrane-bound TLR1/2/4/6 monomers and TLR2 heterodimers. *p < 0.05, **p < 0.01, and ***p < 0.001.

To determine whether the EV71 capsid protein can activate innate immunity via TLR4 and TLR2/TLR4 heterodimer, we first determined the levels of IL-8 in recombinant EV71 capsid protein (VP1, VP2, VP3, and VP4)-stimulated HEK293 cells, in which TLR4 and TLR2/TLR4 heterodimer were overexpressed. IL-8 levels were significantly upregulated in all groups (**Figure 6A**). TLR4 and TLR2/TLR4 heterodimer were co-transfected with EV71 capsid plasmids into UT-SCC-60B cells, and IL-8 levels were found to be upregulated in all groups, except the TLR2/TLR4-EV71VP3 group (**Figure 6B**). *PI3K/AKT* and *MAPK* pathways were also activated at different levels in TLR2-, TLR4-, and TLR2/TLR4-overexpressing HEK293 cells stimulated with recombinant EV71 capsid proteins (VP1, VP2, VP3, and VP4; **Figure 6C**) or in TLR4 and TLR2/TLR4 heterodimer with EV71 capsid protein-overexpressing UT-SCC-60B cells (**Figure 6D**). These results indicate that EV71 capsid proteins activate innate immunity via TLR4 and TLR2/TLR4 heterodimer.

## 4 Discussion

Innate antiviral immunity is the first line of defense against viral replication. Determining the EV71-induced activation mechanism of the innate immune system in host cells can provide insights into virus–host interactions and the pathogenesis of EV71 infection. The specific mechanism of activation of the host innate immune response during EV71 infection remains unknown. EV71-infected patients with HFMD, herpangina, severe brainstem encephalitis, and other manifestations associated with the central nervous system exhibit different proinflammatory responses, such as increased levels of IL-6, IL-8, and other cytokines (27, 43–47). Furthermore, EV71-infected host cells exhibit a robust IFN response (24, 48) and excessive pro-inflammatory cytokine responses (24, 25). Neonatal mice infected with EV71 exhibit high levels of IL-6 with severe tissue damage and high mortality (49). These studies indicate that the host exerts normal immune responses against EV71, which shows immune evasion strategies to increase its replication and spread. Several reviews have summarized the effects of innate immunity on EV71 and the immune evasion process of EV71 (50–53). EV71 mainly depends on its 2A and 3C proteases to cleave PRRs and immune-associated signal molecules to disrupt cellular signal transduction and suppress the production of cytokines and IFN-α/β and IFN-stimulated gene expression. However, the mechanisms involved in the activation of cytokine responses in EV71-infected patients and host cells remain ambiguous. One potential mechanism for activating the innate immunity of host cells against EV71, especially during the early phase of EV71 infection, has been reported but not yet validated.

In this study, we systematically evaluated the roles of cell membrane-bound TLR monomers (TLR1, TLR2, TLR4, and TLR6) and TLR2 heterodimer (TLR2/TLR1, TLR2/TLR6, TLR2/TLR4, and TLR2/CD14) in the inhibition of EV71 replication and activation of innate immunity. We found that EV71 replication was significantly inhibited by TLR monomers and TLR2 heterodimer, and the cellular *PI3K/AKT* and *MAPK* signaling pathways were activated, inducing the production of IL-8. Moreover, EV71 capsid proteins (VP1, VP2, VP3, and VP4) activated TLR monomers (TLR2 and TLR4) and TLR2 heterodimer (TLR2/TLR1, TLR2/TLR6, and TLR2/TLR4), leading to the activation of innate immunity. These results indicate that EV71 and its capsid proteins activate innate immunity, which is necessary for TLR monomer and TLR2 heterodimer recognition, as well as the innate cytokine signaling cascade during the early phase of EV71 infection (**Figure 6E**).

To the best of our knowledge, this study is the first to investigate the mechanism of EV71 infection and replication in detail. Specific roles of cell membrane-bound TLR1/2/4/6 monomers and TLR2 heterodimer in recognizing EV71 and activating innate immunity have not yet been fully elucidated. Currently, the roles of TLRs, particularly cell membrane-bound TLRs in activating innate immunity against EV71 remain unclear. Transcriptional levels of TLR7 and TLR8 are upregulated in EV71-infected host cells (24, 25, 54), indicating that the EV71 genome ssRNA is recognized by TLR7 and TLR8. However, TLR3 may recognize the dsRNA produced in the EV71 genome replication process because the level of TLR3 is also upregulated (24, 54). These TLRs mainly recognize viral RNA and are located in intracellular endosomes; however, whether cell membrane-bound TLR1/2/4/6 monomers and TLR2 heterodimer engage in recognizing EV71 requires further investigation. Humans vaccinated with inactivated EV71 showed altered host innate and adaptive response gene expression (55), and EV71 virus-like particles (VLPs) induced the production of IL-12 and IL-10 via TLR4 (56). Additionally, the expression of TLR2 is significantly upregulated by EV71 and UV-inactivated EV71 in the early phase of infection (25). Our previous study also confirmed that the transcriptional level of TLR2 was upregulated using a transcriptomic sequencing method, and that overexpression of TLR2 and TLR2 heterodimer or activation of TLR2 heterodimer by their ligands significantly inhibited EV71 replication (40). These results indicate that cell membrane-bound TLR2, TLR4, and TLR2 heterodimer play a pivotal role in mediating the innate immune response activation induced by EV71 and that EV71 capsid proteins may be recognized by TLR2, TLR4, and TLR2 heterodimer; however, the underlying mechanisms require further investigation.

To determine the roles of cell membrane-bound TLR monomers (TLR1, TLR2, TLR4, and TLR6) and TLR2 heterodimer (TLR2/TLR1, TLR2/TLR6, TLR2/TLR4, and TLR2/CD14) directly sensing EV71 and contributing to activate innate immunity to limit EV71 replication, we set two strategies to determine the roles of these TLR monomers and TLR2 heterodimer. The first strategy was to test whether normal TLR monomers (TLR1, TLR2, and TLR6) and TLR2 heterodimer (TLR2/TLR1 and TLR2/TLR6) derived from humans or mice could inhibit EV71 replication. Our data demonstrated that EV71 replication was significantly inhibited in two types of host cells overexpressing TLR1, TLR2, TLR6, TLR2/TLR1, and TLR2/TLR6, and increased production of IL-8. To ensure the accuracy of the results, we carried out a second strategy using human–mouse chimeric TLR2 heterodimer (mTLR2/TLR1, TLR2/mTLR1, mTLR2/TLR6, and TLR2/mTLR6) or DN-TLR2 heterodimer (DN-TLR2/TLR1, TLR2/DN-TLR1, DN-TLR2/TLR6, and TLR2/DN-TLR6). Furthermore, the roles of DN-TLRs (DN-TLR1, DN-TLR2, and DN-TLR6) were also evaluated. Our data showed that EV71 replication was also inhibited by human–mouse chimeric TLR2 heterodimer and DN-TLR2 heterodimer; however, DN-TLRs lost their inhibitory ability against EV71. These results are in accordance with those of our earlier investigation (40) and support the idea that both human and mouse TLR1/2/6 monomers and TLR2 heterodimer play important roles in the protection of host cells against EV71. Several viral proteins are recognized by TLR2, including RSV G protein recognized by TLR2/TLR6 heterodimer (36), HIV structural protein p17 and gp41 recognized by TLR2/TLR1 heterodimer, HIV p24 recognized by TLR2/TLR6 heterodimer (34), hepatitis C virus (HCV) core and NS3 recognized by TLR2/TLR1 or TLR2/TLR6 heterodimer (57, 58), and rotavirus NSP4-activated proinflammatory cytokine response recognized by TLR2 (59). Taken together, these results suggest that cell-surface TLR2 and TLR2 heterodimer participate in the recognition of viruses, resulting in a decrease in viral replication and activation of antiviral innate immunity. Unlike other TLRs, TLR2 interacts with its co-receptors, such as TLR1 and TLR6, to form TLR2 heterodimer (60, 61). However, the roles of other cell membrane-bound TLR monomers (TLR1 and TLR6) in viral replication remain unknown. In this study, we systematically evaluated human and mouse TLR1, TLR6, DN-TLR2 heterodimer (DN-TLR2/TLR1 and DN-TLR2/TLR6), and DN-TLRs (DN-TLR1 and DN-TLR6) and found that different TLR monomers (TLR1 and TLR6) and DN-TLR2 heterodimer can inhibit EV71 replication, but not DN-TLR1 and DN-TLR6. These results further confirmed that cell surface TLR1 and TLR6 monomers exhibit anti-EV71 effects while facilitating the formation of TLR2 heterodimer.

TLR4 is another cell membrane-bound TLR that recognizes viral proteins to activate innate immunity. RSV activates innate immunity via TLR4, and RSV F protein is recognized by TLR4 (62, 63). Influenza virus hemagglutinin interacts with TLR4 to induce Janus tyrosine kinase-3 activation (64). Extracellular nucleoproteins of the influenza virus interact with TLR2 and TLR4 to induce the production of IL-1β and IL-6 (37). HIV gp120 binds to TLR2 and TLR4 to activate the nuclear factor (*NF*)-*κB* pathway for the production of TNF-α and IL-8 (35). These studies indicate that TLR4 monomers recognize viral proteins to activate innate immunity and also interact with TLR2. Further studies have confirmed that TLR2 interacts with TLR4 to form the TLR2/TLR4 heterodimer (65). Myeloid differentiation primary response gene 88 mediates TLR2/TLR4 heterodimerization (41). Here, we determined the roles of TLR4 and TLR2/TLR4 heterodimer in EV71 infection. Both human and mouse TLR4 and TLR2/TLR4 heterodimer significantly inhibited EV71 replication, and human–mouse chimeric mTLR2/TLR4 and TLR2/mTLR4 also exhibited inhibitory effects on EV71. DN-TLR4 did not show any inhibitory effect, but DN-TLR2/TLR4 and TLR2/DN-TLR4 inhibited EV71 replication. These results indicate that TLR2 and TLR4 monomers limit EV71 replication. Three single nucleotide mutations in TLR4 (A896G, C1196T, and C2141A) also inhibited EV71 replication, but their inhibition rates of EV71 were significantly decreased compared with those of wild-type TLR4. These results suggest these three nucleotides as functional sites of TLR4.

EV71 is a naked virus consisting of four structural proteins (VP1, VP2, VP3, and VP4) forming a promoter. Five promoters are assembled into a pentamer, and 12 pentamers are assembled to form a capsid with an icosahedral structure (12). Unlike enveloped viruses, such as HIV, which use the envelope glycoprotein gp120, a protein attached to its viral receptor CD4 interacts with cell membrane-bound TLR2 and TLR4 (35). In the life cycle of EV71, especially in the attachment and uncoating phases, EV71 capsid is the first to attach to cell surface molecules, such as viral receptors (SCARB2 and PSGL-1) and other receptors (cell membrane-bound TLR1/2/4/6). EV71 capsid proteins VP1, VP2, and VP3 are exposed on the surface of the capsid, whereas VP4 is located in the internal capsid. Whether EV71 capsid proteins are recognized by cell membrane-bound TLR monomers and TLR2 heterodimer remains unknown. In this study, we mainly focused on TLR2 and TLR4 monomers and TLR2 heterodimer (TLR2/TLR1, TLR2/TLR6, and TLR2/TLR4). We first confirm the findings of our previous study that the levels of TLR2 are upregulated in the UT-SCC-60B cell model. Cells were then stimulated with four recombinant or overexpressed EV71 capsid proteins via plasmid transfection. Concentrations of IL-6 and IL-8 were significantly upregulated and the expression levels of TLR1, TLR2, and TLR6 were upregulated in EV71 capsid protein-overexpressing cells. We confirmed that EV71 capsid proteins induce cytokine production and activate the *PI3K/AKT* and *MAPK* pathways. Well-known functions of EV71 include EV71 VP1-mediated receptor-binding (14, 15) and virulence determination (66, 67). Four capsids containing different neutralizing epitopes can be used as candidate EV71 vaccine epitopes (12, 68). Taken together, our results indicate that EV71 capsid proteins play important roles in innate immune activation via cell membrane-bound TLR2, TLR4, and TLR2 heterodimer. Recently study shows interferon-inducible poly(ADP-ribose) polymerase 9 (PARP9) as the host restriction factor to sense viral RNA and employs PI3K/AKT3 pathway to produce type I interferon (69), and our results also find PI3K/AKT pathway is activated by EV71 and its capsid proteins via TLR2, TLR4, and TLR2 heterodimer. Whether TLR2, TLR4, and TLR2 heterodimer could activate PARP9 mediated PI3K/AKT pathway to induce the phosphorylation of IRF3 and IRF7 to induce type I interferon for inhibiting EV71 replication is still need further investigation.

In summary, our results revealed that cell membrane-bound TLR monomers (TLR1/2/4/6) and TLR2 heterodimer (TLR2/TLR1, TLR2/TLR6, and TLR2/TLR4) recognize EV71, resulting in the activation of innate responses and inhibition of EV71 replication. Moreover, EV71 capsid proteins activated innate immunity via TLR2, TLR4, and TLR2 heterodimer (TLR2/TLR1, TLR2/TLR6, and TLR2/TLR4). Collectively, our results indicated that EV71 capsid proteins, as novel PAMPs, are recognized by membrane-bound TLR monomers and TLR2 heterodimer, improving our understanding of the innate recognition of EV71.

## Data availability statement

The original contributions presented in the study are included in the article/**Supplementary Material**. Further inquiries can be directed to the corresponding author.

## Author contributions

G-CX and L-YD conceived and designed this study. P-PS, DL, MS, and QR performed the cell biology experiments. W-PG, L-YD, and J-LW performed the data analysis. G-CX, P-PS, and L-YD were responsible for writing and the critical reading of the manuscript. All authors contributed to the article and approved the submitted version.
