# Supplementary figures and images for: Cell membrane-bound toll-like receptor-1/2/4/6 monomers and -2 heterodimer inhibit enterovirus 71 replication by activating the antiviral innate response
Source: Front Immunol. 2023 May 3;14:1187035. doi: 10.3389/fimmu.2023.1187035 (PMC10189127; doi:10.3389/fimmu.2023.1187035)

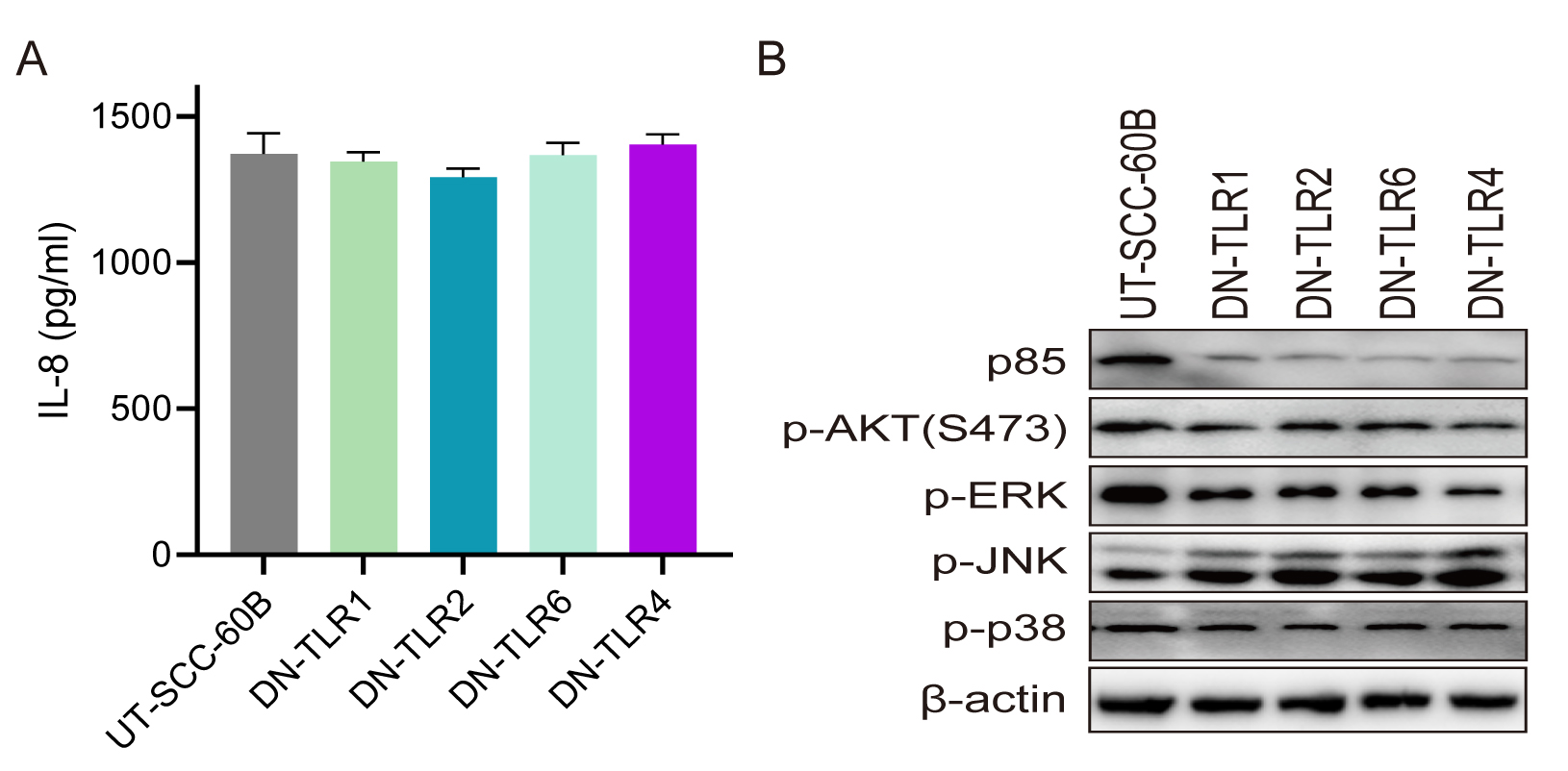

Supplement: Supplementary file 1 [file Image_1.jpeg]
