# Supplementary material for: Cell membrane-bound toll-like receptor-1/2/4/6 monomers and -2 heterodimer inhibit enterovirus 71 replication by activating the antiviral innate response
Source: Front Immunol. 2023 May 3;14:1187035. doi: 10.3389/fimmu.2023.1187035 (PMC10189127; doi:10.3389/fimmu.2023.1187035)
Supplement: Supplementary file 2 [file Table_1.docx]

Supplementary Material

# Supplementary Material

## Supplementary Figures

**Figure S1. Single dominant-negative TIR-less toll-like receptors (TLRs) inhibit cytokine production and signaling pathway activation.** Single dominant-negative TIR-less TLRs (DN-TLR1, DN-TLR2, DN-TLR6, and DN-TLR4) were transfected into UT-SCC-60B cells at a dose of 1 μg for 24 h, followed by infection with EV71 at a multiplicity of infection (MOI) of 1 for 24 h. n = 3. (**A**) Concentrations of interleukin (IL)-8. (**B**) Activation of the phosphoinositide 3-kinase/protein kinase B (*PI3K/AKT*) and mitogen-activated protein kinase (*MAPK*) pathways. *p < 0.05, **p < 0.01, and ***p < 0.001.
